# Decreased WWOX expression promotes angiogenesis in osteosarcoma

**DOI:** 10.18632/oncotarget.17126

**Published:** 2017-04-15

**Authors:** Jia Wen, Zongchao Xu, Jiazhen Li, Yingqiang Zhang, Wenzhe Fan, Yu Wang, Mingjian Lu, Jiaping Li

**Affiliations:** ^1^ Department of Interventional Oncology, The First Affiliated Hospital of Sun Yat-sen University, Guangzhou, Guangdong, 510080, The People’s Republic of China; ^2^ Emergency Department, The First Affiliated Hospital of Zhengzhou University, Zhengzhou, Henan, 450052, The People’s Republic of China; ^3^ Department of Orthopedics, The First Affiliated Hospital of Zhengzhou University, Zhengzhou, Henan, 450052, The People’s Republic of China

**Keywords:** osteosarcoma, WWOX, angiogenesis, RUNX2, bcl-2

## Abstract

WWOX (WW domain-containing oxidoreductase) is known to be an important tumor suppressor in cancer. In this study, we used samples from 201 osteosarcoma patients to investigate the effects of WWOX on angiogenesis and invasion. WWOX levels were negatively correlated with RUNX2 and VEGF levels, but were not correlated with OPN levels. Among the clinicopathological characteristics examined, WWOX was associated only with response to neoadjuvant chemotherapy, and its expression in osteosarcoma tissues was a predictor of disease-free survival. WWOX promoted apoptosis and inhibited invasion and expression of bcl-2, OPN, RUNX2, and VEGF in osteosarcoma cells *in vitro*. In MG-63 cells, bcl-2 increased VEGF expression, while RUNX2 increased VEGF and OPN expression. Administration of DNA methylation inhibitors increased WWOX expression in MG-63 cells and methylation of WWOX gene promoter CpG island in the osteosarcoma of patients was associated with suppression of WWOX expression. Overexpression of WWOX in osteosarcoma cells inhibited tube formation in co-cultured HUVEC cells, and high WWOX expression was associated with decreased microvessel density (MVD). These results suggest that reduced WWOX expression in osteosarcoma inhibits apoptosis, promotes invasion and increases MVD.

## INTRODUCTION

Osteosarcoma, which is highly malignant and invasive, is frequently seen in teenagers. Pulmonary metastasis occurs in early stages in osteosarcoma patients and is associated with a poor prognosis [1]. Although surgery and chemotherapy have substantially improved the prognosis of osteosarcoma [2], high rates of postoperative multidrug resistance usually lead to recurrence and metastasis [3].

Angiogenesis plays an essential role in the growth, invasion, and metastasis of osteosarcoma [4]. It has been shown that drugs such as sorafenib can repair abnormal blood vessels and improve the transportation of oxygen and drugs to tumor cells, thus increasing the efficacy of chemotherapy [5]. Therefore, it could be an approach to treat osteosarcoma by developing drugs inhibiting angiogenesis. Critical factors in the process of angiogenesis may be potential targets for therapy in osteosarcoma patients.

WW domain-containing oxidoreductase (WWOX), which is encoded by the chromosomal fragile site spanning gene [6], is a tumor suppressor in several human cancers [7–9]. It is likely inactivated by translocations [10, 11], loss of heterozygosity [12], copy number aberrations [13], and promoter hypermethylation [14]. WWOX protein might interact with several molecules that are associated with tumor progression and angiogenesis, including RUNX2 [13, 15], bcl-2 [16, 17], P73 [18], c-Jun [19] , and Dvl-2 [20]. In a previous study, periosteal osteosarcoma relapse occurred along the diaphysis in 31% of adolescent WWOX knockout mice [21]. Additionally, WWOX immunoreactivity was reduced in 58% of osteosarcoma specimens, while WWOX expression was strong in all the normal bone specimens [22]. Furthermore, overexpression of WWOX inhibited the metastasis of human osteosarcoma and reduced tumor sizes in a nude mouse model [23]. These findings suggest that WWOX plays a role in angiogenesis and invasion in human osteosarcoma.

In this study, we investigated the role of WWOX in angiogenesis in human osteosarcoma and its effects on the expression of CD34, RUNX2, and VEGF to understand their potential interaction in the development of osteosarcoma.

## RESULTS

### WWOX expression was associated with RNUX2 and VEGF expression and microvessel density

**Figure 1:**
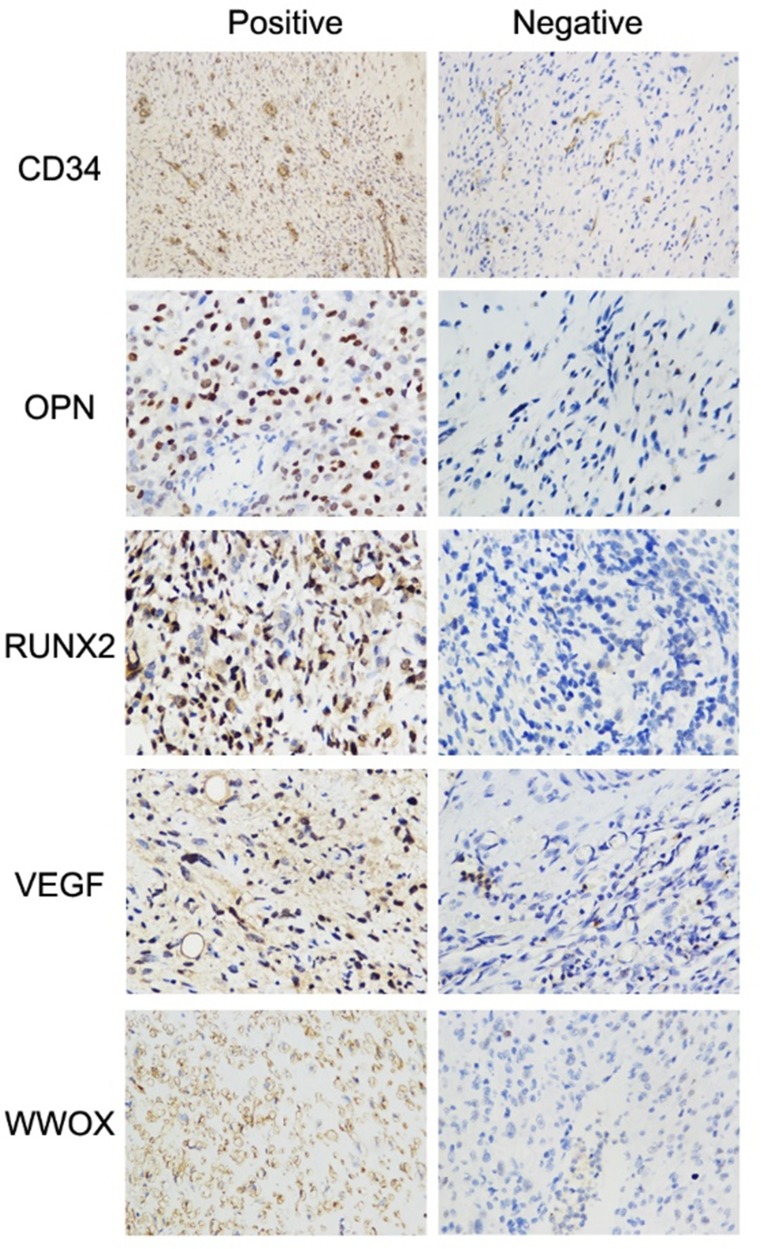
Representative immunohistochemical staining of WWOX, VEGF, RUNX2, OPN, and CD34 in osteosarcoma specimens Images for WWOX, VEGF, RUNX2, and OPN were taken under 400× magnification, and for CD34 were taken under 200 × magnification.

We quantified WWOX, RNUX2,VEGF, and OPN expression using immunohistochemistry scores. WWOX expression was negatively correlated with RUNX2 (r = –0.172, *P* = 0.015) and VEGF (r = –0.142, *P* = 0.044) expression. The correlations were weak although significant. There was no correlation between WWOX expression and OPN expression (r = –0.103, *P* = 0.147) (Table 1, Figure 1). We then examined MVD via immunohistochemical staining for CD34 and evaluated its association with WWOX expression. High WWOX expression was associated with decreased MVD (*P* = 0.026). The correlation was weak although significant (Table 1, Figure 1). Finally, association between WWOX expression and response to neoadjuvant chemotherapy in osteosarcoma patients was analysed by Spearman correlation with processing the response to chemotherapy as a hierarchical data PR, SD and PD and were progressively included. The results showed that WWOX expression was associated with response to neoadjuvant chemotherapy but not with gender, age, or tumor position (Table 2).

**Table 1:** Associations between WWOX expression and RNUX2/VEGF/OPN expression and MVD in osteosarcoma patients

WWOX	*n*	RNUX2				VEGF				OPN				MVD
		**-**	**+**	**++**	**+++**	**-**	**+**	**++**	**+++**	**-**	**+**	**++**	**+++**	
-	95	34	19	26	16	16	19	40	20	43	32	19	1	28.78 ± 7.06
+	49	19	8	14	8	11	8	21	9	25	13	10	1	28.80 ± 5.81
++	29	18	8	1	2	8	5	11	5	20	5	4	0	28.03 ± 5.17
+++	28	16	3	4	5	8	8	10	2	12	15	1	0	25.11 ± 5.75
r		–0.172				–0.142				–0.103				0.026#
P		0.015*				0.044*				0.147*				

*Spearman’s correlation analysis. #, ANOVA.

**Table 2:** Associations between WWOX expression and clinicopathological characteristics

Clinicopathological characteristics		WWOX expression				*P* value (Spearman correlation)
		Negative	Weak positive	Positive	Strong positive	
Gender	Male	62	31	18	17	0.624
	Female	33	18	11	11	0.624
Age (years)		26.2 ± 15.6	23.6 ± 13.5	27.2 ± 14.2	25 ± 13.5	0.916
Primary site	Limbs	70	37	25	23	0.182
	Other	25	12	4	5	
Response to neoadjuvant chemotherapy	PR	10	4	5	4	0.025
	SD	19	19	7	8	
	PD	30	8	4	5	

Abbreviations: PR, partial response; SD, stable disease; PD, progressive disease.

### WWOX expression is predictive of disease-free survival in osteosarcoma patients

**Figure 2:**
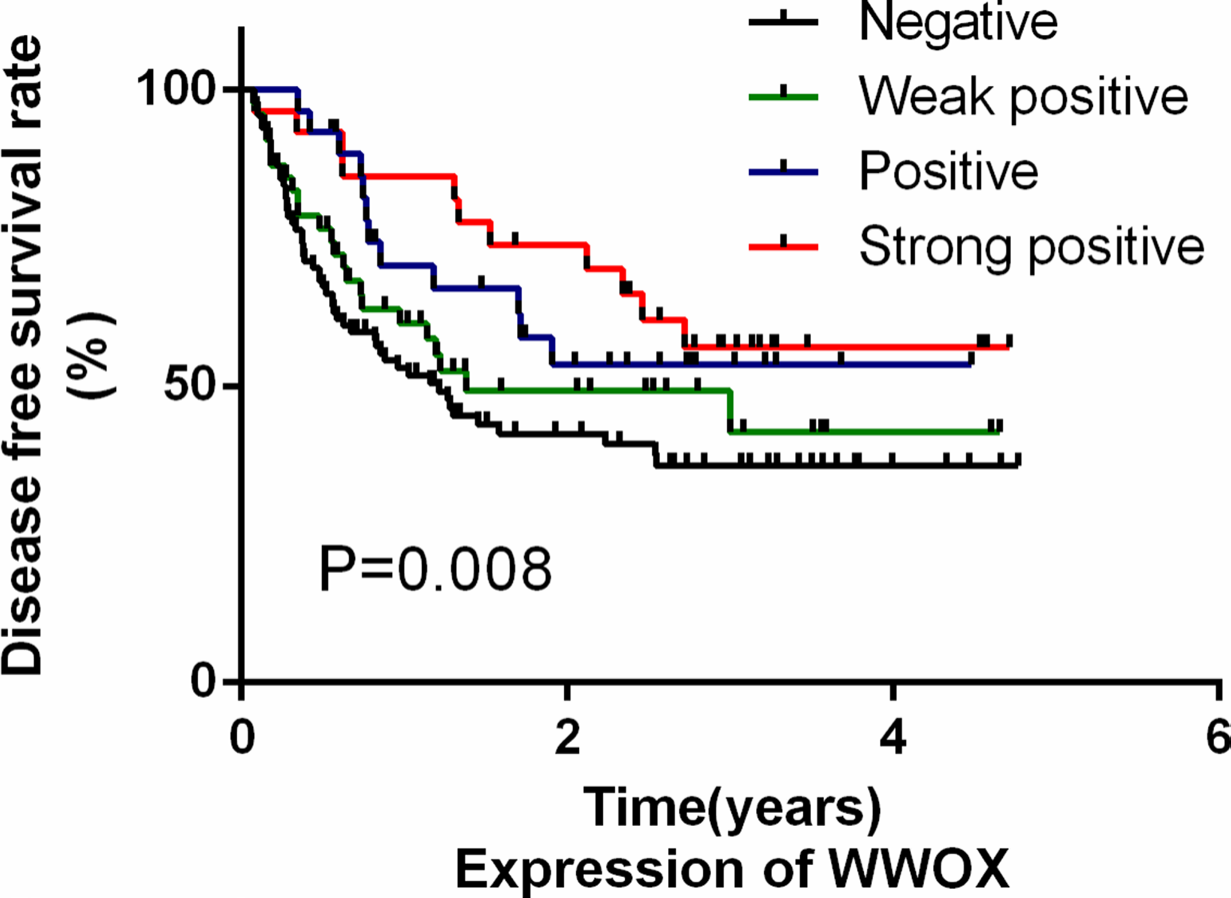
WWOX expression in osteosarcoma tissues was a predictor of disease-free survival in osteosarcoma patients Kaplan-Meier survival analysis was performed using WWOX IHC scores and tumor-free survival times.

We then examined whether WWOX expression in osteosarcoma tissues was predictive of disease-free survival (DFS) in osteosarcoma patients using Cox regression analysis. Because response to neoadjuvant chemotherapy, which is a classic predictor of DFS in osteosarcoma patients, might mask the effects of other factors, we did not include that measure in the analysis. Univariate Cox analysis identified low WWOX expression, high VEGF expression, and high MVD as poor prognostic factors for DFS (*P* < 0.05; Table 3); age, gender, and RUNX2 and OPN expression were not associated with DFS. We then used a multivariate Cox proportional hazard model to examine whether low WWOX expression was a prognostic factor for DFS independent of other risk factors including age, gender, MVD, and VEGF, OPN, and runx2 expression. Low WWOX expression was an independent predictor for poor DFS in T2N0M0 osteosarcoma patients (Table 3); patients with lower WWOX expression had lower DFS rates, while patients with higher WWOX expression had higher DFS rates (Figure 2). Similar results were also observed for VEGF expression; no other factors were independent prognostic markers for DFS.

**Table 3:** Cox regression analysis of independent factors for disease free survival

Factor	Univariate cox analysis		Multivariate cox analysis	
	HR (95% CI)	*P* value	HR (95% CI)	*P* value
Gender		0.770		0.798
Male	1		1	
Female	1.063 (0.705–1.604)		0.974 (0.625–1.435)	
WWOX expression		0.015		0.031
negative	1		1	
positive	0.613 (0.413–0.909)		0.639 (0.425–0.960)	
VEGF expression		0.010		0.025
negative	1		1	
positive	2.102 (1.194–3.700)		1.947 (1.089–3.481)	
OPN expression		0.348		0.251
negative	1		1	
positive	1.219 (0.806–1.845)		1.278 (0.841–1.942)	
RUNX2 expression		0.254		0.986
negative	1		1	
positive	1.263 (0.846–1.884)		1.004 (0.660–1.527)	
age	1.001 (0.988–1.015)	0.844	1.004 (0.990–1.081)	0.579
MVD	1.034 (1.001–1.068)	0.044	1.026 (0.993–1.061)	0.126

### WWOX promoted apoptosis in osteosarcoma cells

**Figure 3:**
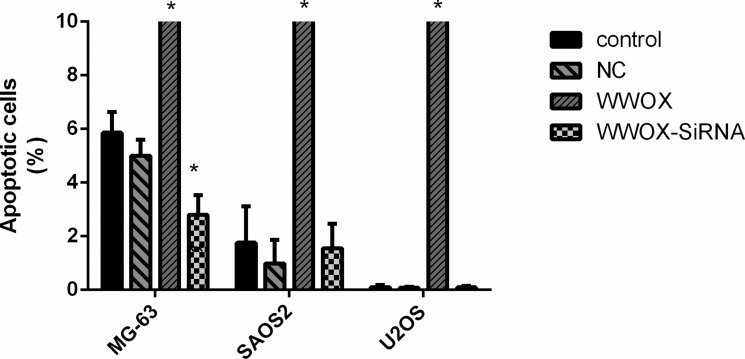
Overexpression of WWOX promoted apoptosis in osteosarcoma cells U2OS, SAOS2, and MG-63 cells were transfected with WWOX overexpression plasmid, WWOX-siRNA, or control constructs followed by 48 h of incubation. The cells were collected and stained with FITC-labeled annexin V and propidium iodide, after which apoptosis rates were measured using flow cytometry. NC: negative control. In this group, the cells were transfected with blank vector plasmids. *P < 0.05. Bars depict the mean ± SD from three replicates in an independent experiment. The results shown are representative from three repeated experiments

To determine whether WWOX plays a role in the survival of osteosarcoma cells, we overexpressed WWOX and knocked down WWOX expression in U2OS, SAOS2, and MG-63 cells and examined apoptosis rates using flow cytometry. Overexpression of WWOX increased apoptosis in all osteosarcoma cells, while WWOX knockdown decreased apoptosis in MG-63 cells, compared to the normal and negative controls (Figure 3). These results suggest that WWOX promoted apoptosis in osteosarcoma cells.

### WWOX inhibited the invasion of osteosarcoma cells

**Figure 4:**
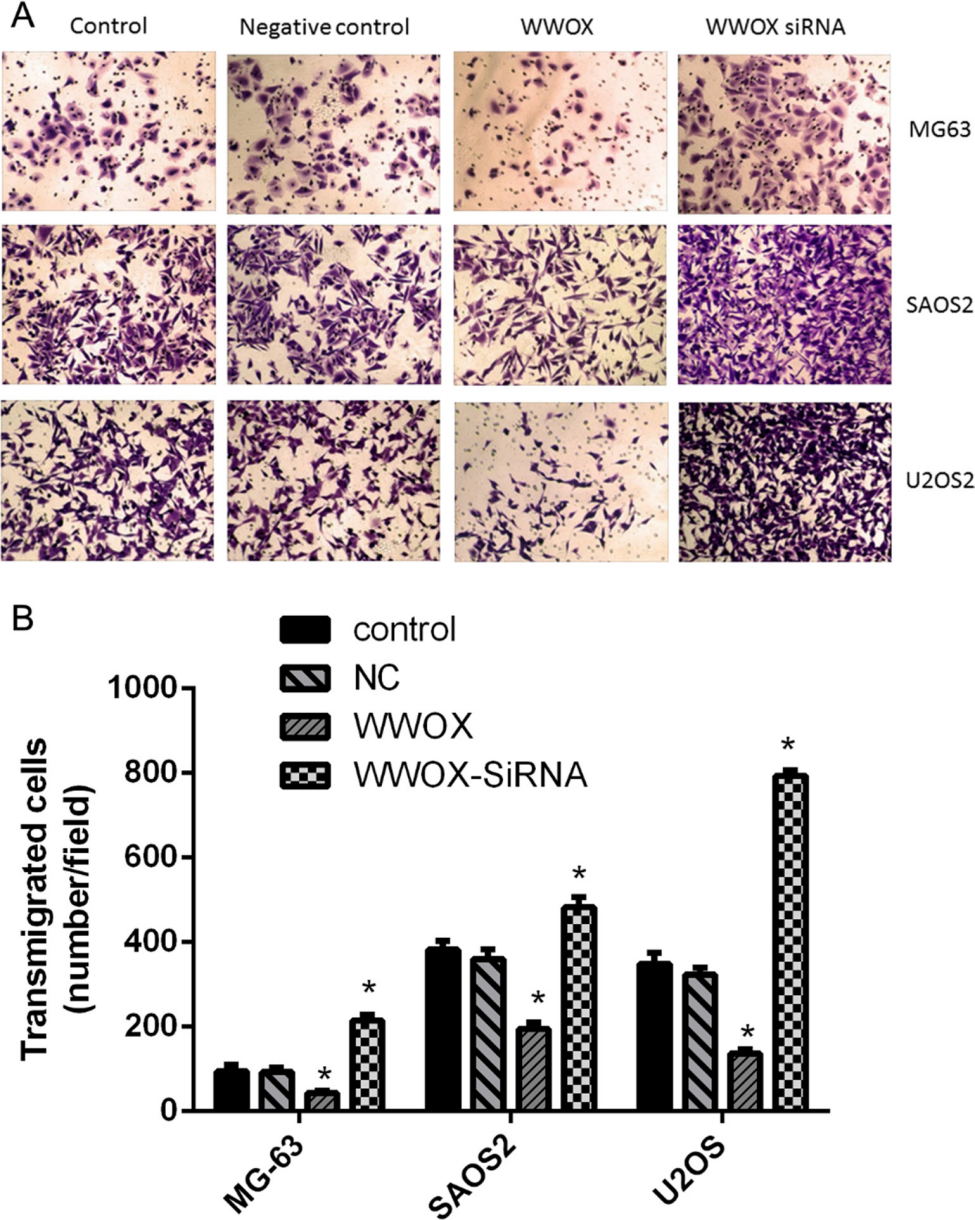
WWOX inhibited the invasion of osteosarcoma cells Invasion assays were performed in osteosarcoma cells using Boyden chambers. NC: negative control. In this group, the cells were transfected with blank vector plasmid. Images were taken under 100× magnification. *P < 0.05. Bars depict the mean ± SD from three replicates in an independent experiment. The results shown are representative from three repeated experiments.

To determine whether WWOX plays a role in osteosarcoma cell invasion, we examined the invasion capacity of U2OS, SAOS2, and MG-63 cells with WWOX overexpression or knockdown using the Boyden chamber assay. WWOX overexpression decreased, while WWOX knockdown increased, the numbers of migrated osteosarcoma cells compared to the normal and negative controls (Figure 4). These results suggest that WWOX inhibited the invasion of osteosarcoma cells.

### WWOX inhibited bcl-2, RUNX2, VEGF, and OPN in osteosarcoma cells

**Figure 5:**
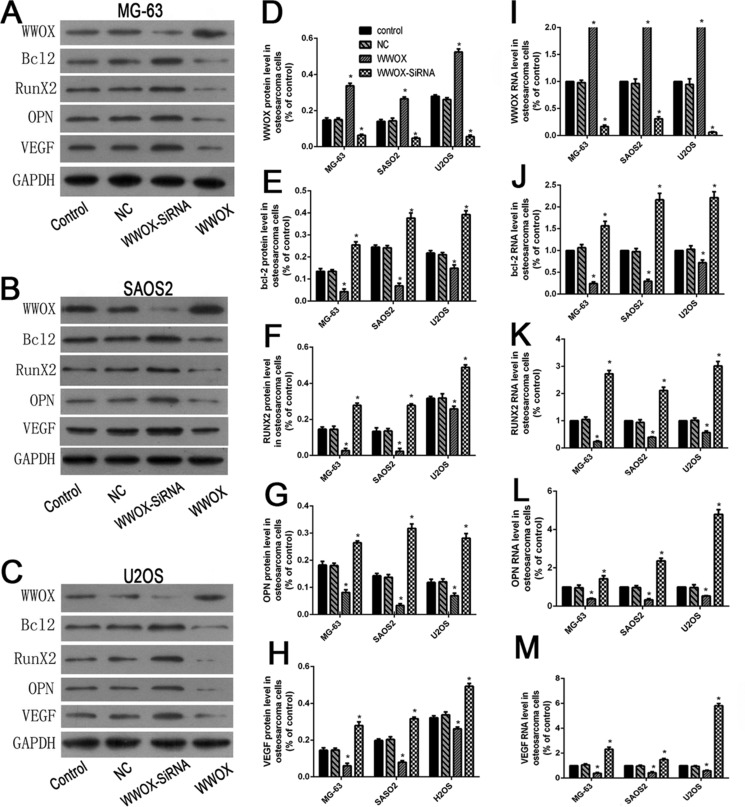
WWOX inhibited bcl-2, RUNX2, VEGF, and OPN expression in osteosarcoma cells (**A–C**) WWOX, bcl-2, RUNX2, VEGF, and OPN protein expression in MG63 (A), SAOS2 (B), and U2OS (C) cells with WWOX overexpression or knockdown. Protein levels were determined using Western blots. (**D–H**) Quantification of the results in A-C normalized to GAPDH protein levels. (**I–M**) Relative WWOX, bcl-2, OPN, RUNX2, and VEGF mRNA levels in MG63, SAOS2, and U2OS cells with WWOX overexpression or knockdown. mRNA expression was determined using qRT-PCR and normalized to GAPDH mRNA levels. NC: negative control. In this group, the cells were transfected with blank vector plasmid. *P < 0.05. Bars depict the mean ± SD from three replicates in an independent experiment. The results shown are representative from three repeated experiments.

To determine whether WWOX regulates bcl-2, RUNX2, VEGF, and OPN expression in osteosarcoma cells, we used Western blotting and qRT-PCR to examine protein and mRNA levels of these factors in U2OS, SAOS2, and MG-63 cells with WWOX overexpression or knockdown. WWOX overexpression decreased, while WWOX knockdown increased, RUNX2, bcl-2, VEGF, and OPN protein and mRNA levels in osteosarcoma cells compared to the normal and negative controls (Figure 5). These results suggest that WWOX inhibited bcl-2, RUNX2, VEGF, and OPN expression in osteosarcoma cells.

### Regulatory cascade involving WWOX, bcl-2, OPN, RUNX2, and VEGF in MG-63 cells

**Figure 6:**
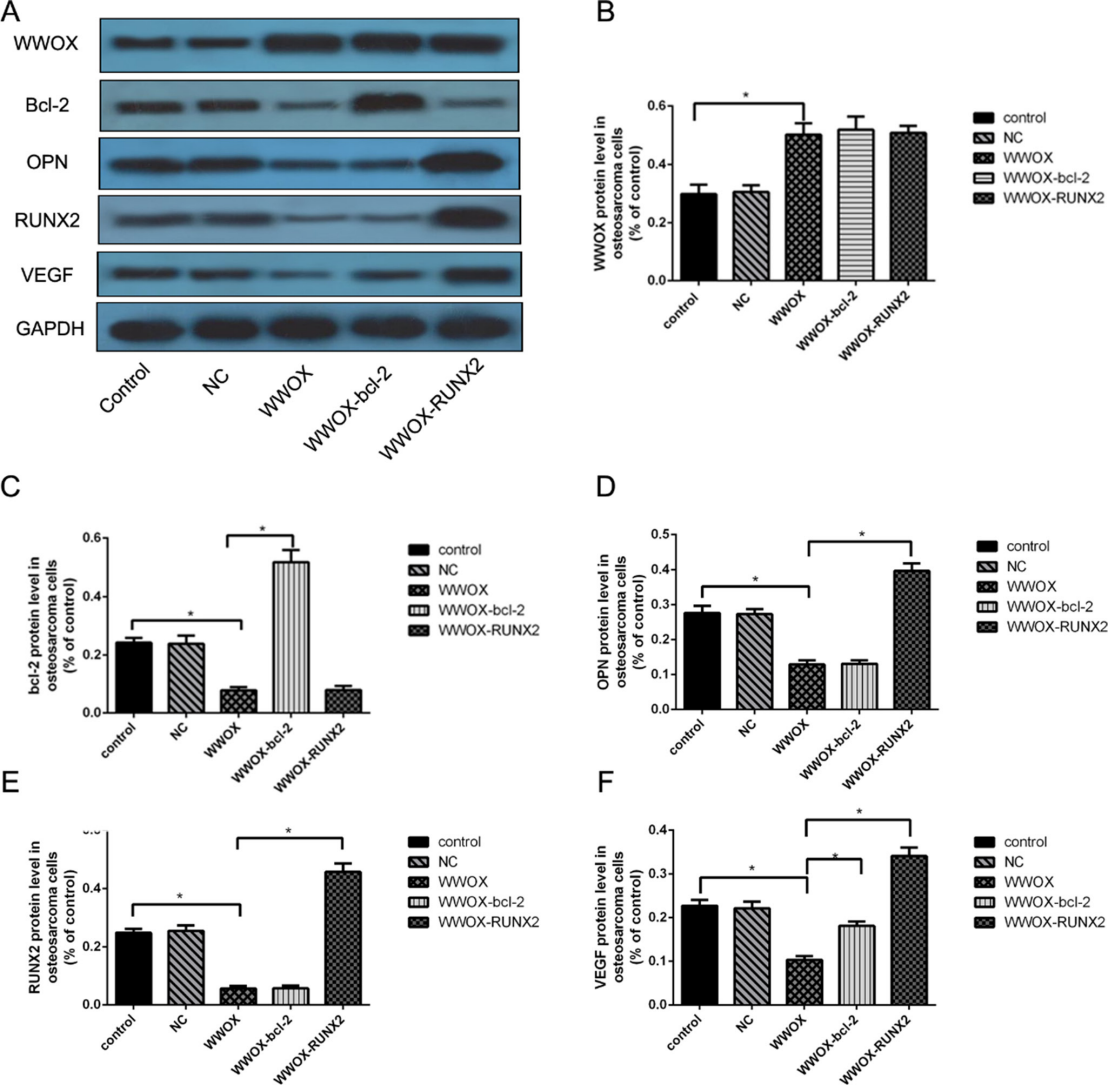
Regulatory cascade involving WWOX, bcl-2, OPN, RUNX2, and VEGF in MG-63 cells (**A**) WWOX, bcl-2, RUNX2, VEGF, and OPN protein expression in MG-63 cells overexpressing WWOX, bcl-2, and/or RUNX2 were determined using Western blotting. (**B–F**) Quantification of the results in (A) after normalization to GAPDH protein levels. NC: negative control. In this group, the cells were transfected with blank vector plasmids. *P < 0.05. Bars depict the mean ± SD from three replicates in an independent experiment. The results shown are representative from three repeated experiments.

To characterize the regulatory cascade involving WWOX, bcl-2, OPN, RUNX2, and VEGF in osteosarcoma cells, we transfected MG-63 cells overexpressing WWOX with bcl-2 or RUNX2 expression plasmids and measured WWOX, bcl-2, OPN, RUNX2, and VEGF expression using Western blotting. WWOX overexpression decreased RUNX2, bcl-2, VEGF, and OPN protein levels in MG-63 cells compared to the normal and negative controls (Figure 6A, 6C–6F). WWOX and bcl-2 overexpression together did not change WWOX (Figure 6A, 6B), OPN (Figure 6A, 6D), or RUNX2 (Figure 6A, 6E) protein levels, but did increase VEGF protein levels (Figure 6A, 6F), compared to WWOX overexpression alone in MG-63 cells (Figure 6A, 6C–6F). WWOX and RUNX2 overexpression together did not change WWOX (Figure 6A, 6B) or bcl-2 (Figure 6A, 6C) protein levels, but did increase OPN (Figure 6A, 6D) and VEGF (Figure 6A, 6F) levels, in MG-63 cells compared to WWOX overexpression alone. These results suggest that WWOX inhibited bcl-2, OPN, RUNX2, and VEGF expression, while bcl-2 increased VEGF expression and RUNX2 increased VEGF and OPN expression.

### WWOX expression was suppressed by DNA methylation

**Figure 7:**
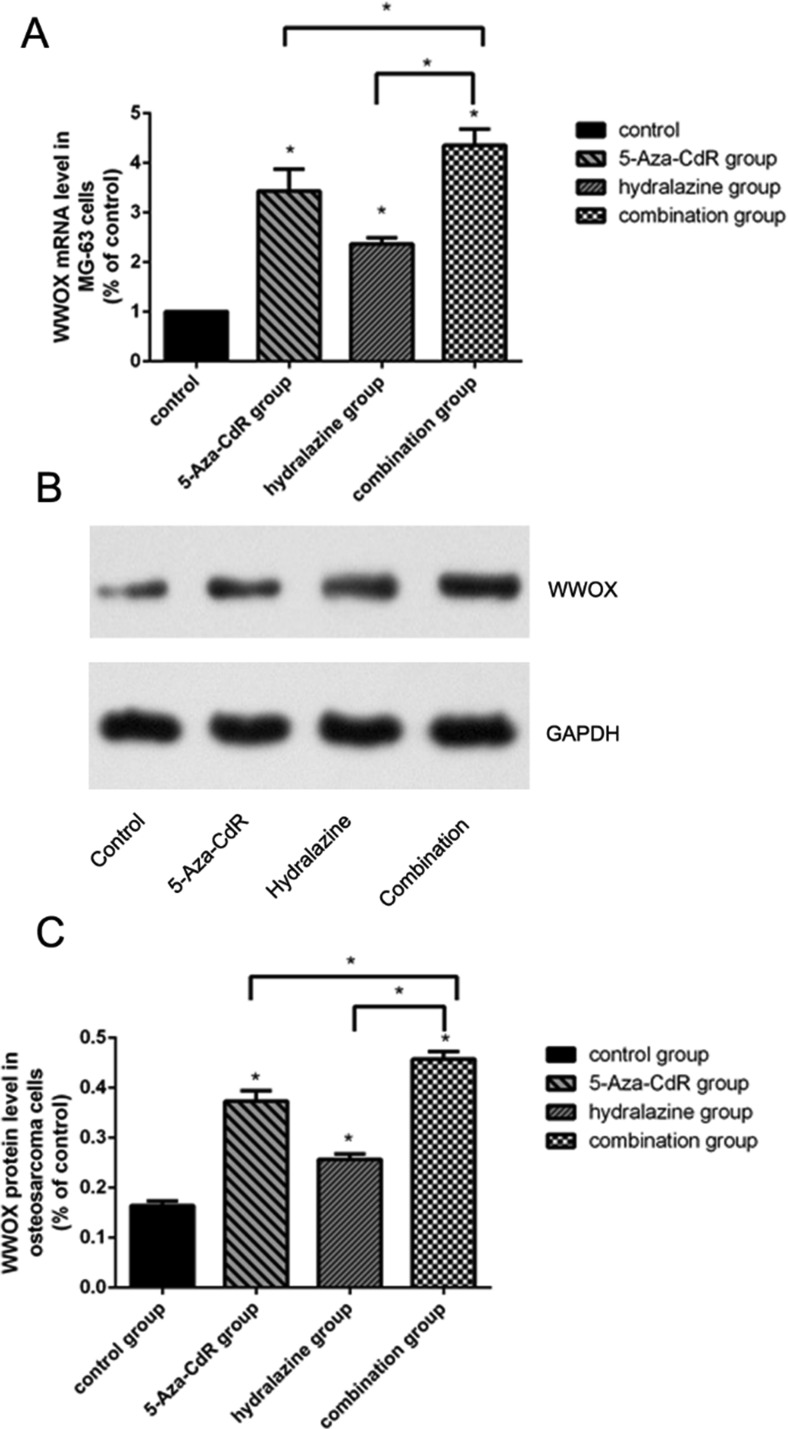
DNA methylation inhibitors increased WWOX expression in MG-63 cells (**A**) Relative WWOX mRNA levels in MG-63 cells treated with hydralazine (1.0 μmol/L) and 5-Aza-CdR (10.0 μmol/L) alone or in combination. mRNA expression was determined by qRT-PCR and normalized to GAPDH mRNA levels. (B) WWOX protein expression in MG-63 cells treated with hydralazine (1.0 μmol/L) and 5-Aza-CdR (10.0 μmol/L) alone or in combination were determined using Western blotting. (**C**) Quantification of the results in (**B**) after normalization to GAPDH protein levels. *P < 0.05. Bars depict the mean ± SD from all samples in a group in an independent experiment. The results shown are representative from three repeated experiments.

**Figure 8:**
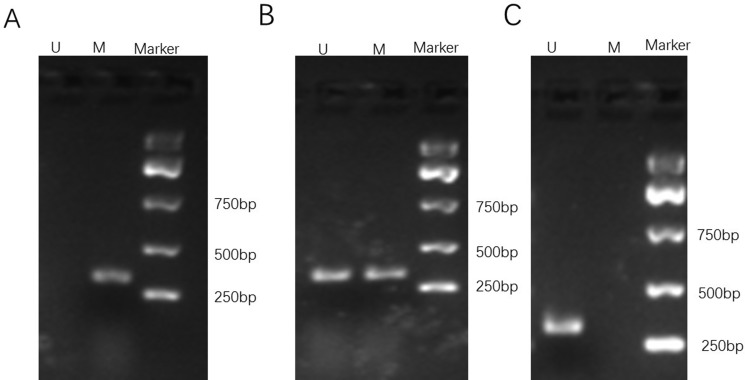
Representative gel electrophoresis of PCR products for methylation assay of the WWOX gene promoter CpG islands in osteosarcoma of patients (**A**) completely methylated; (**B**) partly methylated; (**C**) unmethylated. M, methylated band; U, unmethylated band.

To determine whether WWOX expression is epigenetically regulated by DNA methylation in osteosarcoma cells, we treated MG-63 cells with the DNA methylation inhibitors hydralazine and 5-Aza-CdR alone or in combination and measured WWOX mRNA and protein levels using qRT-PCR and Western blotting. The results showed that treatment with hydralazine, 5-Aza-CdR, or both increased WWOX mRNA levels (Figure 7A) and protein levels (Figure 7B, 7C) in MG-63 cells. We further performed a methylation status analysis of WWOX gene promoter CpG island in the osteosarcoma of patients and found that methylation of WWOX gene promoter CpG island in the osteosarcoma of patients was associated with suppression of WWOX expression (Figure 8, Table 4). These results suggest that DNA methylation inhibitors increased, while DNA methylation suppressed WWOX expression in osteosarcoma

**Table 4:** Associations between WWOX expression and methylation status of WWOX gene promoter CpG island

Methylation status	WWOX expression			
	**Negative**	**Weak Positive**	**Positive**	**Strong Positive**
Completely Methylated	13	3	2	1
Partly Methylated	4	1	1	3
Unmethylated	5	2	2	5

Spearman’s correlation coefficients: r = –0.350, *P* = 0.023.

### WWOX overexpression in osteosarcoma cells inhibited tube formation in co-cultured HUVEC cells

**Figure 9:**
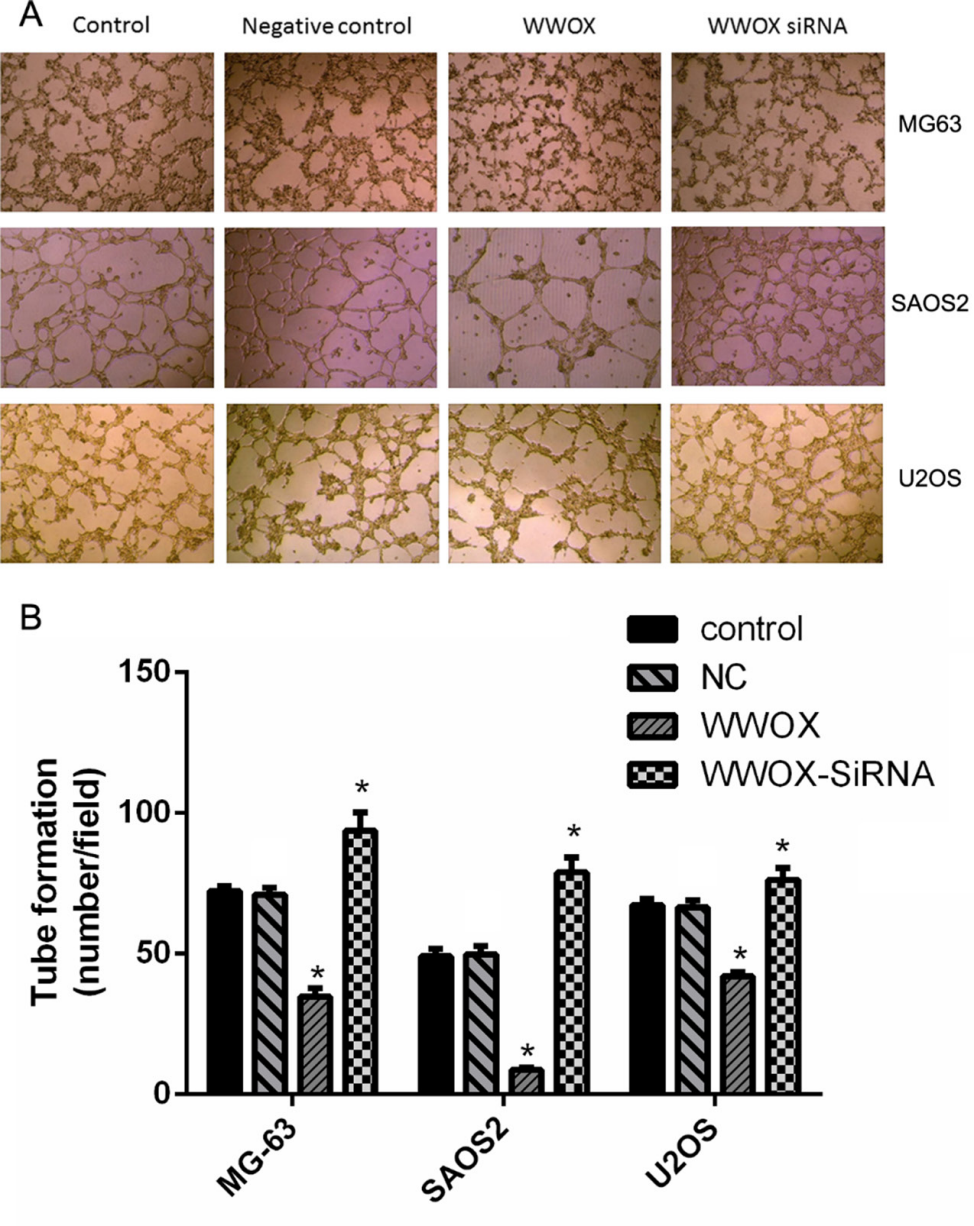
WWOX inhibits tube formation in HUVECs Images were taken under 40× magnification. *P < 0.05. NC: negative control. In this group, the cells were transfected with blank vector plasmid. Bars depict the mean ± SD from three replicates in an independent experiment. The results shown are representative from three repeated experiments.

To determine whether WWOX plays a role in angiogenesis, tube formation was examined in HUVEC cells co-cultured with U2OS, SAOS2, and MG-63 osteosarcoma cells with WWOX overexpression or knockdown. WWOX overexpression in the osteosarcoma cells decreased, while WWOX knockdown in the osteosarcoma cells increased, tube formation in the HUVEC cells compared to the normal and negative controls (Figure 9). These results suggest that WWOX inhibited tube formation in HUVEC cells.

## DISCUSSION

**Figure 10:**
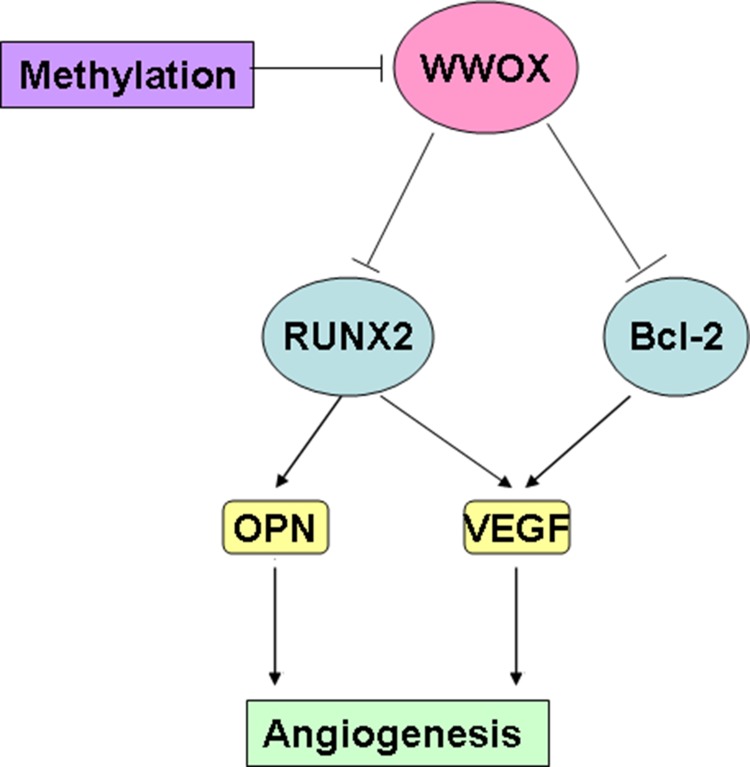
WWOX inhibits angiogenesis in osteosarcoma -- a proposed model

In this study, we investigated whether and the underlying mechanisms by which WWOX affects angiogenesis and invasion in osteosarcoma. We found that WWOX levels were negatively correlated with RUNX2 and VEGF levels, but were not correlated with OPN levels. In addition, although WWOX expression was associated with response to neoadjuvant chemotherapy in osteosarcoma patients, it was not associated with other clinicopathological characteristics, including gender, age, or tumor position. WWOX expression in osteosarcoma tissues was also a predictor of disease-free survival (DFS) in osteosarcoma patients. WWOX promoted apoptosis in and inhibited the invasion of osteosarcoma cells. In addition, WWOX inhibited the expression of bcl-2, OPN, RUNX2, and VEGF in osteosarcoma cells, while bcl-2 increased VEGF expression and RUNX2 increased VEGF and OPN expression in MG-63 cells. Furthermore, inhibition of DNA methylation increased WWOX expression in MG-63 cells. Overexpression of WWOX in osteosarcoma cells inhibited tube formation in co-cultured HUVEC cells, and high WWOX expression was associated with decreased MVD. Together, these results suggest that reduced WWOX expression in osteosarcoma cells inhibited apoptosis, promoted invasion, upregulated bcl-2, OPN, RUNX2, and VEGF expression, and increased microvessel density (MVD) (Figure 10). WWOX may therefore inhibit angiogenesis and invasion in osteosarcoma.

Our finding that administration of DNA methylation inhibitors increased WWOX expression in MG-63 cells is consistent with previous studies [24, 25]. We further found that methylation of WWOX gene promoter CpG island in the osteosarcoma of patients is associated with suppression of WWOX expression. While other factors such as abnormal gene copy number, can also reduce WWOX expression [13], our data suggest that DNA methylation is likely a key contributor to reduced WWOX expression in osteosarcoma cells.

In this study, we also found that WWOX promoted apoptosis in osteosarcoma cells, which is consistent with the results of previous studies [8, 14, 16]. WWOX expression differs among cell lines with varying degrees of tumorigenicity and metastasis [26]. Additionally, the sensitivity of WWOX expression to a specific siRNA may differ among cell lines; U2OS and SAOS2 cells appeared to be much less sensitive to siRNA than MG-63 cells. This might partially explain why siRNA-mediated WWOX knockdown decreased apoptosis rates in MG-63 cells, but not in U2OS or SAOS2 cells. In contrast, overexpression of WWOX increased apoptosis rates in all three cell lines.

Our data also showed that WWOX inhibited the invasion of osteosarcoma cells. This is consistent with the work of Del Mare and Aqeilan [23], which showed that WWOX-induced inhibition of invasion in osteosarcoma cells was mediated by RUNX2. We confirmed that WWOX inhibited RUNX2 expression in osteosarcoma cells. Furthermore, we showed that WWOX also inhibited the expression of bcl-2, OPN, and VEGF in osteosarcoma cells. WWOX levels were also negatively correlated with RUNX2 and VEGF levels, but not with OPN levels. In addition, bcl-2 increased the expression of VEGF, but not OPN or WWOX, and RUNX2 increased the expression of VEGF and OPN, but not bcl-2 or WWOX, in MG-63 cells. These results suggest that WWOX is an upstream regulator of bcl-2 and RUNX2, which in turn regulate the downstream factors and possible effectors OPN and VEGF (Figure 9). These data also suggest that WWOX-mediated bcl-2 and RUNX2 independently regulate VEGF (Figure 9). Whether VEGF and OPN expression is directly regulated by WWOX remains to be investigated.

Angiogenesis plays a critical role in cancer progression by promoting tumor metastasis, which results in poor prognoses [27–29]. In this study, we showed for the first time that WWOX expression was negatively associated with MVD and confirmed that WWOX overexpression in osteosarcoma cells inhibited tube formation in co-cultured HUVEC cells. These data suggest that decreased WWOX expression might promote angiogenesis in osteosarcoma.

VEGF and OPN, which play central roles in angiogenesis in almost all types of tumors, including osteosarcoma [30, 31], are targets of many anti-angiogenetic drugs [32, 33]. Bcl-2 has anti-apoptotic effects in a variety of tumors [34, 35], including osteosarcoma [36–38]. In addition, bcl-2 promotes the expression of genes necessary for angiogenesis and VEGF expression in hypoxic tumor cells [17], implying that bcl-2 might promote angiogenesis as well. RUNX2 is a master regulator of cell cycle-related factors in osteoblast progenitor cells [39] and in osteosarcoma [40]. In addition, RUNX2 can promote the expression of genes that are necessary for angiogenesis [15]. Previous studies showed that bcl-2 expression was inhibited by WWOX in breast cancer [16] and bladder cancer [41], and that RUNX2 expression is up-regulated in WWOX-deficient mice [26]. Here, we found that WWOX inhibited the expression of RUNX2, bcl-2, OPN, and VEGF in osteosarcoma cells. It is possible that WWOX inhibits the expression of these factors similarly in many cell types in addition to osteosarcoma and breast cancer cells. Furthermore, decreased WWOX expression promoted angiogenesis in osteosarcoma by upregulating these same genes.

Although WWOX is sensitive to hormones [42], we did not find any associations between WWOX expression and gender. WWOX expression was not associated with age or tumor position in osteosarcoma patients either. However, we did find an association between WWOX expression and response to neoadjuvant chemotherapy. Neoadjuvant chemotherapy is an effective approach for treating osteosarcoma [2], and resistance to chemotherapy often leads to poor prognosis [43]. This association may help explain our finding that WWOX expression in osteosarcoma tissues was a predictor of disease-free survival in osteosarcoma patients.

In conclusion, we found that the inhibition of WWOX expression, which was due at least in part to DNA methylation, inhibited apoptosis, promoted invasion, upregulated bcl-2, OPN, RUNX2, and VEGF expression, and increased MVD in osteosarcoma cells. These findings elucidate the underlying mechanisms by which decreased WWOX levels promote angiogenesis and invasion in osteosarcoma.

## MATERIALS AND METHODS

### Patients

This study was approved by the Ethics Committee of Sun Yet-sen University and Zhengzhou University. Patients diagnosed with osteosarcoma (*n* = 201) at the First Affiliated Hospital of Sun Yet-sen University and Zhengzhou University between 2011 and 2015 based on pathological examination of paraffin-embedded tissue specimens were included in the study. Written informed consent was obtained from all patients. Clinical and histopathological patient characteristics are listed in Table 5. All patients had a clinical stage of T2N0M0, and none of the patients received radiotherapy prior to surgery. Disease-free survival was monitored during follow-ups, and the median follow-up time for all 201 patients was 29.5 months.

**Table 5:** Clinicopathological characteristics of osteosarcoma patients

Characteristics		Cases	%
Gender	Male	128	63.7
	Female	73	36.3
Age (years)	< 15	47	23.4
	15–20	60	29.9
	21–30	32	15.9
	31–40	19	9.4
	> 40	43	21.4
Tumor position	Limbs	155	77.1
	Other	46	22.9
Neoadjuvant chemotherapy	Evaluated	123	61.2
	No data available	50	24.9
	Not administered	28	13.9
Radical excision	Yes	196	97.5
	No	5	2.5
Recurrence/metastasis	Yes	100	51
	No	96	49
Outcome	Alive	137	67.2
	No follow-up data	27	13.4
	Death	39	19.4

### Immunohistochemistry

WWOX, VEGF, OPN, and RUNX2 expression were determined using immunohistochemical staining as previously described [13]. Antibodies against WWOX, VEGF, and RUNX2 were purchased from Abcam (Cambridge, UK) and used at dilutions of 1:500, 1:100, and 1:100, respectively. PBS was used as a negative control for all IHC. Immunostaining was examined under a light microscope by two pathologists blinded to all patient data. Ten fields (400×) that included >100 cells were randomly examined for each section. Staining intensity was scored based on staining color: a score of 0 indicated no staining, 1 indicated yellow staining, 2 indicated tan staining, and 3 indicated brown staining. The extent of staining was also scored based on the proportion of positive tumor cells: < 5% positive tumor cells was scored 0, 6‒25% was scored 1, 26‒50% was scored 2, 51‒75% was scored 3, and > 75% was scored 4. IHC scores were calculated by multiplying the staining intensity and extent scores and were used to categorize protein expression as follows: an IHC score of 0 was considered negative (“–”), scores of 1–3 were considered weak positive (“ + “), scores of 4–7 were considered moderate positive (“++”), and scores of 8–12 were considered strong positive (“+++”).

### Microvessel density assay

Microvessel density (MVD) was assessed by IHC with a CD34 antibody (BioGenex Life Sciences, 1:100) as described in a previous report [44]. Numbers of microvessels were counted in 10 randomly-selected fields for each normal bone and osteosarcoma tissue using a light microscope (OLYMPUS CKX41 U-CTR30-2, Japan). The average number of microvessels was calculated for each sample and was defined as the microvessel density (MVD).

### Cell culture and transfection

The U2OS, SAOS2, and MG-63 human osteosarcoma cell lines were purchased from the Shanghai Cell Bank of the Chinese Academy of Sciences (Shanghai, China) and cultured in DMEM supplemented with 10% fetal bovine serum (Gibco, USA), 5 µg of streptomycin/mL, and 50 U penicillin/mL at 37°C in a humidified incubator with 5% CO_2_. Once they reached 30–50% confluence, the osteosarcoma cells were transfected with bcl-2 or RUNX2 expression plasmid, Lenti-WWOX, Lenti-WWOX siRNA, and Lenti-EV using Lipofectamine® 2000 transfection reagent (Invitrogen, USA) according to the manufacturer’s protocol. Untransfected cells served as normal controls, and cells transfected with Lenti-EV served as negative controls. The WWOX, bcl-2, and RUNX2 expression plasmids and WWOX-siRNA were purchased from Shanghai Genechem Co. Ltd. (Shanghai, China). The WWOX siRNA sequences were 5′-CCA AGU CCA UGC AAC AGG Gtt-3′ (sense) and 5′-CCC UGU UGC AUG GAC UUG Gtt -3′ (anti-sense).

### Flow cytometry

Apoptosis was examined in human osteosarcoma cells collected 48 h after transfection using flow cytometry with the Annexin V-FITC/PI Apoptosis kit according to the manufacturer’s protocols and a FACS Calibur flow cytometer (Becton Dickinson, USA) with CellQuest software. More than 5×10^5^ cells were examined for each sample, and the experiment was independently repeated three times.

### Boyden chamber assay

The invasion capacity of human osteosarcoma cells was assessed using a Boyden chamber assay. Transwell chambers with a pore size of 8 μm were pre-coated with matrigel. A total of 1 ×10^5^ cells in 100 μL of serum-free DMEM were seeded into the upper chambers, and 600 μL of DMEM with 10 % FBS was added to the lower chamber. After incubation at 37°C for 24 h, the invaded cells were fixed in 4% paraformaldehyde (Beyotime Institute of Biotechnology, China). After 30 min of fixation, the cells were stained with crystal violet for 10 min. Numbers of invaded cells were counted in five randomly selected fields for each well. All experiments were independently performed three times.

### Quantitative RT-PCR assay

Total RNA was isolated from cells using Trizol reagent (Invitrogen, USA) according to the manufacturer’s instructions. 1.0 μg of RNA from each sample was reverse-transcribed into cDNA and subjected to real-time PCR using SYBR® Green PCR Master Mix (TOYOBO). The primers used for real-time PCR were as follows: WWOX forward 5′-GGA CCC AAG ACT GGC GTT TA-3′, reverse 5′-GTG ACC ACA ACC ACT TTG CC-3′; RUNX2 forward 5′-TTA CCC CTC CTA CCT GAG CC-3′, reverse 5′-CCT AGG CAC ATC GGT GAT GG-3′; bcl-2 forward 5′-GAC TTC GCC GAG ATG TCC AG-3′, reverse 5′-CAA TCC TCC CCC AGT TCA CC-3′; VEGF forward 5′-ACT GCC ATC CAA TCG AGA CC-3′, reverse 5′-CTC CAG GCC CTC GTC ATT G-3′; OPN forward 5′-TCC CTC GAT GTC ATC CCT GT-3′, reverse 5′-CCC TTT CCG TTG TTG TCC TG-3′; GAPDH forward 5′-ACC CAG AAG ACT GTG GAT GG-3′, reverse, 5′-TCT AGA CGG CAG GTC AGG TC-3′. GAPDH mRNA level was used as a reference for the other genes, and the relative mRNA levels of the treatment groups were compared to those of the controls. All experiments were independently performed three times.

### Western blotting analysis

Total proteins were extracted from cells using RIPA buffer with PMSF (Thermo, USA), and the protein concentration was determined using the BCA method (Beyotime, China). Equal amounts of protein were resolved by sodium dodecyl-polyacrylamide gel electrophoresis and then transferred onto polyvinylidene fluoride (PVDF) membranes (Millipore, USA). The membranes were blocked with 5% non-fat milk at room temperature for 1 h and then incubated with primary antibodies (WWOX, RUNX2, bcl-2, OPN, or VEGF) overnight at 4°C. After three washes, the membranes were incubated with horseradish peroxidase-conjugated secondary antibody (Beyotime) for 1 h at 37°C. The bands containing the target proteins were then detected using the enhanced chemiluminescence (ECL) kit (Millipore, USA).

### DNA methylation assay

DNA samples were extracted from the decalcified osteosarcoma tissue samples (about 30mg) according to the protocol of the Ezup column animal genome DNA Extraction Kit (Shanghai Shengong Bioengineering Co, Ltd China). The concentration of DNA was determined using UV spectrophotometry and the DNA samples with A260/A280:1.8–2.0 were used for DNA methylation assay according to the protocol of the CpGenomeTM Fast DNA Modification bisulfite Kit (Chemicon company, USA). MSP primers were synthesized by Guangzhou Youdi biotech Co. Ltd. The primer sequences for the methylated WWOX gene promoter were: forward 5′-TAT GGG CGT CGT TTT TTT AGT T -3′, and reverse 5′-CAA TCT CCG CAA TAT CGC GAC A -3′. The primer sequences for the unmethylated WWOX gene promoter were: forward 5′-TAT GGG TGT TGT TTT TTT AGT T -3′, and reverse 5′-CAA TCT CCA CAATAT CAC AAC A -3′. 5 μl PCR products were resolved by 1.5% agarose gel electrophoresis analysis, photographed under ultraviolet light. If the promoter was completely methylated, only PCR product from methylation primers was seen. If the promoter was unmethylated, only PCR product from unmethylation primers was seen. If the promoter was partially methylated, both PCR products from methylation primers and unmethylation primers were seen.

### Tube formation assay

The tube formation assay was performed as previously described [45–47] with slight modifications. Briefly, U2OS, SAOS2, or MG-63 osteosarcoma cells (starting density 2 × 10^4^ cell/ml) were been transfected with WWOX overexpression, WWOX siRNA, control plasmids (NC, negative control) or vehicle control for 4–6 h and incubated in serum-free RPMI-1640 (Hyclone, Cat.No.SH30809.01B) medium supplemented with Streptomycin (Hyclone, Cat.No. SH30010) for 72 h. These serum-free conditioned medium was collected and stored at –80°C, and incubated at 37°C before use. 48-well plates were coated with 200 μL of matrigel (BD Biosciences, USA) per well via incubation at 37°C for 2 h. HUVECs were collected from culture, washed with PBS buffer (Hyclone, Cat.No.SH30256.01B), resuspended in conditioned medium in a density 1 × 10^5^ cells/ml. Each HUVECs sample was added onto the matrigel at a density of 2 × 10^4^ cells/well and incubated for 4–6 h. Three fields were randomly selected and examined for tubes using an inverted optical microscope. The number of tubes in each field was counted and compared among treatment groups.

### Statistical analysis

All data were analyzed using SPSS 17.0 software (SPSS Inc., Chicago, IL, USA). Student’s *t*-test, ANOVA, Mann-Whitney U test, χ^2^ test, Pearson chi square test, or Spearman rho test were used for statistical analyses. Kaplan-Meier survival analysis was used to evaluate the association between WWOX IHC scores and tumor-free survival time*. P* < 0.05 was considered statistically significant.
